# Transformation of water chemistry in the Stara River under climate and land use changes in the Carpathians

**DOI:** 10.1038/s41598-025-17660-4

**Published:** 2025-10-02

**Authors:** Anna Bojarczuk, Anna Biernacka

**Affiliations:** 1 Institute of Geography and Spatial Management, Jagiellonian University, Gronostajowa 7, Kraków, 30-387 Poland; 2 Doctoral School of Exact and Natural Sciences, Jagiellonian University, prof. S. Łojasiewicza 11, Kraków, 30- 348 Poland

**Keywords:** River water chemistry, Anthropogenic pollution, Climate change impact, Land use change, Biogeochemistry, Climate sciences, Environmental sciences, Hydrology

## Abstract

The chemistry of the Stara River changes along its course due to both natural processes and human activities. Its chemical composition is influenced by tributary inflows, groundwater infiltration, and anthropogenic pollution. Particularly noticeable are sudden changes in the concentrations of nitrogen and phosphorus compounds, as well as Na⁺ and Cl⁻ ions, indicating a strong impact of human activities, especially in agricultural and urbanized areas. Although no significant trends in total precipitation have been recorded, higher temperatures lead to increased evaporation, which results in higher ion concentrations. Climate change accelerates rock weathering, leading to increased ion release, particularly HCO_3_⁻ and Ca²⁺. Land use changes significantly impact water quality. The expansion of urbanized areas leads to water quality deterioration, especially through increased concentrations of nitrogen, phosphorus, and chlorides. In contrast, an increase in forested areas contributes to water quality improvement by reducing erosion and retaining pollutants. In agricultural regions, a decrease in SO_4_^2−^ concentrations is observed due to reduced fertilizer use. PCA analysis indicates that hydrometeorological conditions are the most important factor shaping water chemistry. Land use also plays a significant role – urban areas increase the biogenic load, while agriculture influences NH_4_⁺, NO_2_⁻, and PO_4_^3−^ concentrations.

## Introduction

The quality of river waters results from complex environmental processes influenced by both natural factors and human activities^1^. Among natural factors, climate change, hydrological conditions, and the geochemical properties of the catchment play a crucial role. Changes in precipitation patterns, temperature, and seasonal biological dynamics can significantly affect water chemistry, determining the concentrations of nutrients and pollutants^2^. However, anthropogenic factors, such as land-use changes, agricultural expansion, and urbanization, often lead to rapid changes in the chemical composition of rivers^3^. This results from a combination of local pollution sources, natural self-purification processes, and the presence of riparian vegetation, which can act as a filter for nutrients and contaminants^4^.

Water from mountains is usually of better quality because these areas are generally less exposed to industrial or agricultural activities due to pressures from human activity. However, they are highly sensitive to anthropogenic transformations, which can lead to water quality degradation and disruptions in the functioning of the entire ecosystem^5^. Human activities such as tourism development, urbanization, agriculture, land-use changes, and climate change significantly impact the condition of these waters and their ability to self-purify and regenerate^6^. For example, tourism activities can lead to increased nutrient enrichment and bacterial contamination in river waters, as observed by Lenart-Boroń et al.^7^ in the Białka River. The intensification of agriculture, which is associated with increased use of fertilizers and pesticides, results in the contamination of surface water from pollution from diffuse sources. Non-point source pollution from livestock and poultry farming contributes to elevated levels of total nitrogen (TN) and chemical oxygen demand (COD) in rivers, as observed in the East River and Baoxing River^8^. Meanwhile, climate change (rising air temperatures and changes in precipitation patterns) in mountainous regions reduces water availability and increases the concentration of dissolved organic and inorganic carbon in streams, leading to deteriorating water quality^9^. In mountain lakes and streams, rising temperatures and permafrost thawing cause higher concentrations of dissolved substances such as sulfate and base cations. This happens due to enhanced mineral weathering and increased contact with weatherable minerals^10^. Changes in precipitation patterns, snowmelt timing, and the ratio of rain to snow also significantly affect both water quality and resources^11,12^.

Mountains play a crucial role in global water supply, often referred to as “natural water towers” due to their significant contribution to meeting both natural and anthropogenic water demands. Mountains provide water resources for billions of people worldwide. More than 50% of mountain areas have an essential or supportive role for downstream regions, with 7% of global mountain areas providing essential water resources and another 37% delivering important supportive supply^13^. It is estimated that mountain water is vital for the survival of about 1.4 billion people, particularly in regions such as the Himalayas or the Andes. By the mid-21st century, about 1.5 billion people, or 24% of the world’s lowland population, will be critically dependent on mountain runoff^14^.

In the Polish Carpathians (Southern Poland), most drinking water intakes come from surface waters such as rivers and retention reservoirs. For example, in the Małopolskie Voivodeship, the share of total drinking water abstraction from surface waters in this region is approximately 75%, one of the highest in Poland^15,16^. Due to the mountainous nature of the region and the numerous watercourses, surface waters are a key source of drinking water supply for the population. However, surface water intake presents challenges, including the need to protect these waters and purify them from pollutants originating from various sources, such as industrial activities, agriculture, and municipal contamination. In recent decades, significant changes in land use have occurred in the Carpathian region. There has been a noticeable increase in forested areas, particularly due to the abandonment of agricultural land and natural forest succession. Agricultural land has also significantly decreased, often replaced by forests or grasslands^17^. This trend is evident in various parts of the Carpathians, including the Polish Carpathians and the Apuseni Mountains in Romania, where agricultural activities have declined since the early 1990 s, following the fall of the communist regime^18^. Urbanization of these areas also impacts land use changes, as there has been growing development of residential and recreational areas. For example, in the Polish Carpathians, there has been a shift from residential/agricultural buildings to residential/recreational buildings. Along with urbanization, there also has been strong development of road networks, facilitating access to remote areas and contributing to further land use changes^19^. An additional challenge for water quality in the Carpathians is the observed climate changes, including increasingly frequent extreme events, such as droughts and intense rainfall, which may further affect the availability and quality of surface waters^20–22^.

The aims of the study were to:

1. identify long-term trends in the chemical composition of river water and determine the impact of land-use changes and climate change on these trends.
2. analyze the spatial variability of water chemistry along the river course, identifying areas more sensitive to pollution and assessing the influence of point and diffuse pollution sources.

## Study area

**Fig. 1:**
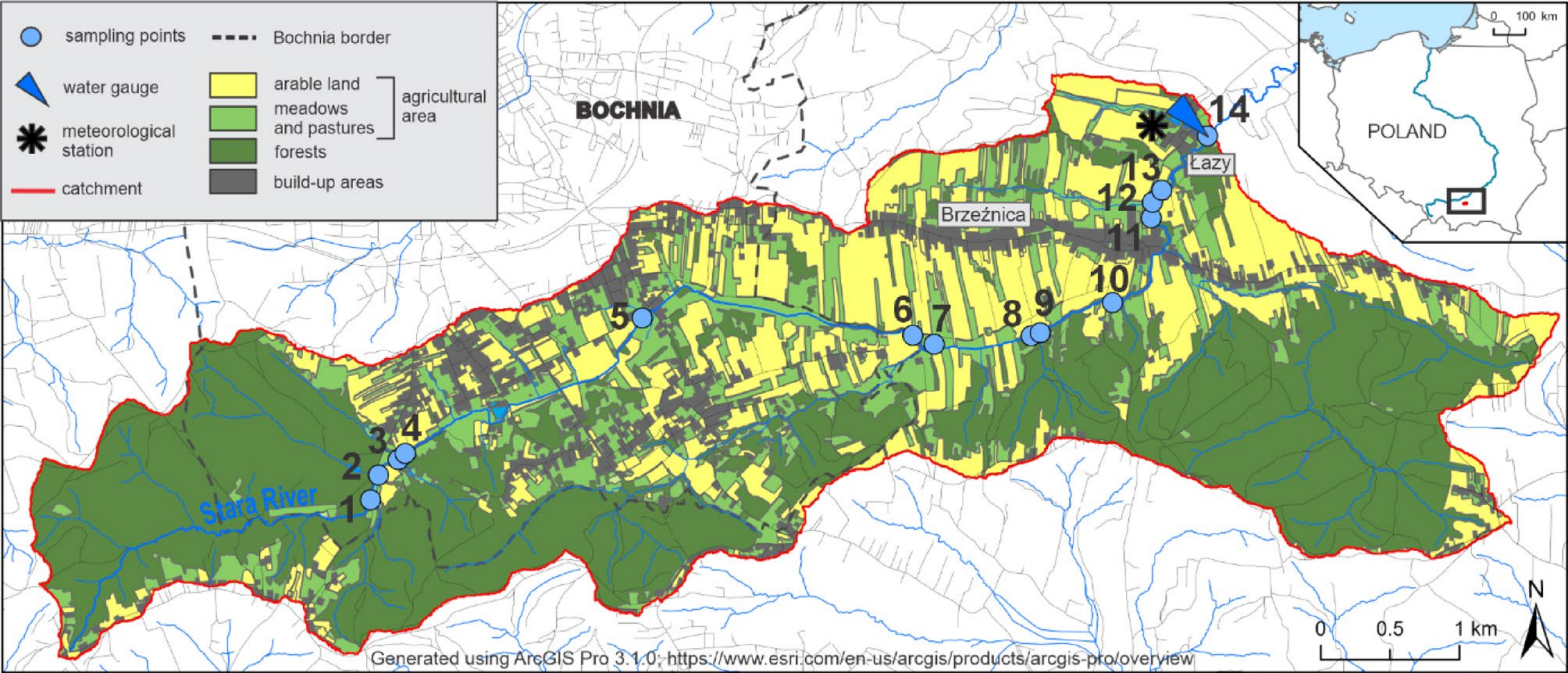
Study area and location of sampling points.

The Stara River catchment (22.3 km²) is located in the Carpathian Foothills (southern Poland) (Fig. 1), which consists of two levels of the Carpathian Foothills threshold. The higher (southern) level is composed of resistant flysch, while the lower (northern) level consists of flysch and Miocene rocks covered by loess-like formations^23^. The predominant soil types in the catchment area (80%) are typical clay-illuvial soils (*Haplic Luvisol*) and stagnogleyic clay-illuvial soils (*Stagnic Luvisol*). The area is also characterized by the presence of brown soils, as well as alluvial and deluvial soils^24,25^. The Stara River catchment is located in a temperate climate zone, within the moderately warm submontane climatic belt^26^. For the study period 2003–2023, the average annual air temperature was 9.3 °C, with an average annual minimum of 4.6 °C and a maximum of 14.4 °C. The mean annual precipitation over the same period was 716 mm. Almost half of the catchment area (mainly the southern and western parts) is covered by forests, consisting mainly of hornbeam, riparian, and beech forests. There are also deciduous and coniferous woodland communities, including fir, pine, and oak^27^. A quarter of the area is occupied by agricultural land. The field layout is characterized by small farms with long, narrow parcels cultivated along the slope of the land. One-fifth of the catchment area consists of pastures and meadows, while almost one-tenth is covered by urban areas (BDOT10k). In the upper part, the study area is partially located within the boundaries of the town of Bochnia. The remaining catchment area Lies within rural and forested regions. In 1984, a Scientific Station of Jagiellonian University was established in the lower part of the catchment. A meteorological station was set up, and the Stara River was equipped with a water gauge. Since 1993, scientists from the station have been conducting hydrological and meteorological monitoring.

## Methods

This article identifies long-term trends using data from 2003 to 2023 on water chemistry and daily discharge of the Stara River, as well as daily meteorological data from the Scientific Station of the Jagiellonian University in Łazy, which is located within the studied catchment. However, for the analysis of the spatial variability of water chemistry along the course of the Stara River (14 sampling points – Fig. 1), data from 2022 to 2024 were used. The selection of sampling sites along the Stara River was based on hydrological and land-use criteria, with particular attention given to locations most likely to reflect the influence of anthropogenic pressure. Monitoring points were strategically situated near the confluences with major tributaries, as well as in the vicinity of known point sources of pollution, such as wastewater discharges, and within or adjacent to agricultural areas. The meteorological data included daily total precipitation (PP), average daily air temperature (AvgTA), minimum air temperature (MinTA), and maximum air temperature (MaxTA). Water chemistry samples were generally collected once per month, resulting in an average of 12 samples per year, with a higher frequency (twice per month) applied until 2008. The water chemistry data comprised parameters such as pH, electrical conductivity (EC), total suspended solids (TSS), water mineralization (TDS), and the concentrations of 11 ions. Major ions (Ca²⁺, Mg²⁺, Na⁺, K⁺, HCO_3_^−^, SO_4_^2−^, Cl⁻) as well as nitrogen and phosphorus compounds (NH₄⁺, NO_3_⁻, NO_2_⁻, PO_4_^3−^) were determined using a DIONEX 2000 ion chromatograph with an AS-4 autosampler. The method and accuracy of chemical analyses were carried out in accordance with the standards: PN-89 C-04638^28^, PN-EN ISO 10304−1^29^, and PN-EN ISO 14911^30^. The detection limits (LOD) for all analyzed ionic species are summarized in Table 1.

**Table 1:** Limits of detection (LOD) for water chemistry parameters in the Stara River.

Parameter	Ca^2+^	Mg^2+^	K^+^	NH_4_^+^	Na^+^	HCO_3_^−^	SO_4_^2−^	NO_3_^−^	NO_2_^−^	Cl^−^	PO_4_^3−^
LOD [mg·dm^−3^]	0.005	0.005	0.005	0.005	0.01	0.025	0.01	0.0025	0.0025	0.0025	0.01

Water mineralization was calculated as the sum of the determined ions, while the total inorganic nitrogen (TIN) concentration was calculated as the sum of NH_4_-N, NO_3_-N, and NO_2_-N concentrations. Total suspended solids (TSS) in water were determined using the gravimetric method.

To illustrate changes in land use, it has been decided that the data will be presented at intervals of five years (2005, 2010, 2015, 2020). Data for 2005 and 2020 was taken from available reliable resources: the calculations made as part of the KNB project from the years 2002–2004^31^ and polish Database of Topographic Objects^32^. The remaining land-use data were derived from the supervised classification of Landsat 5 (2010) and Sentinel-2 (2015) satellite imagery performed with ArcGIS Pro 3.1.0. To assess interannual variability in atmospheric precipitation, a Relative Precipitation Index (RPI) analysis was carried out for the period 2003–2023.

In the study, the Mann-Kendall (MK) method^33,34^ and Sen’s slope estimator^35^ were used to analyze trends in water chemistry, as well as meteorological and hydrological data. Trend calculations were performed both on an annual scale and separately for seasons, as well as for the growing and non-growing seasons. The Mann-Kendall method is one of the most commonly used statistical methods for detecting trends in time series of hydrological, meteorological, and environmental data. It is a non-parametric test, making it particularly useful for analyzing highly variable data and studying long-term trends in water chemistry, temperature, precipitation, and river flows. Sen’s slope estimator, also known as Sen’s method, is a non-parametric technique used to determine the magnitude of trends in time series data. It is often applied alongside the MK test to quantify the rate of change over time in environmental data. In the study, Principal Component Analysis (PCA) and the Kaiser criterion were applied to identify the most important factors shaping the water chemistry of the Stara River. PCA is a widely used multivariate statistical technique that helps identify key factors influencing water chemistry by reducing the dimensionality of complex datasets. This method transforms a set of correlated variables into a smaller number of uncorrelated principal components (PC), which explains the maximum variance within the dataset. The Kaiser Criterion is a widely used method for selecting the most significant principal components in PCA. According to this criterion, only components with an eigenvalue greater than 1 should be retained for further analysis. For PCA, data on water chemistry, discharge, and land use in the Stara River catchment area were used. To examine the relationship between land use and the physicochemical parameters of the Stara River’s water, Pearson correlation analysis was used. The existence of statistically significant relationships was tested at a significance level of *p* = 0.05. The strength of correlation coefficients was interpreted according to the scale proposed by Overholser and Sowinski^36^. Calculations were performed using Statistica 13 software.

## Results

### Spatial variability of water chemistry along the course of the Stara river

**Fig. 2:**
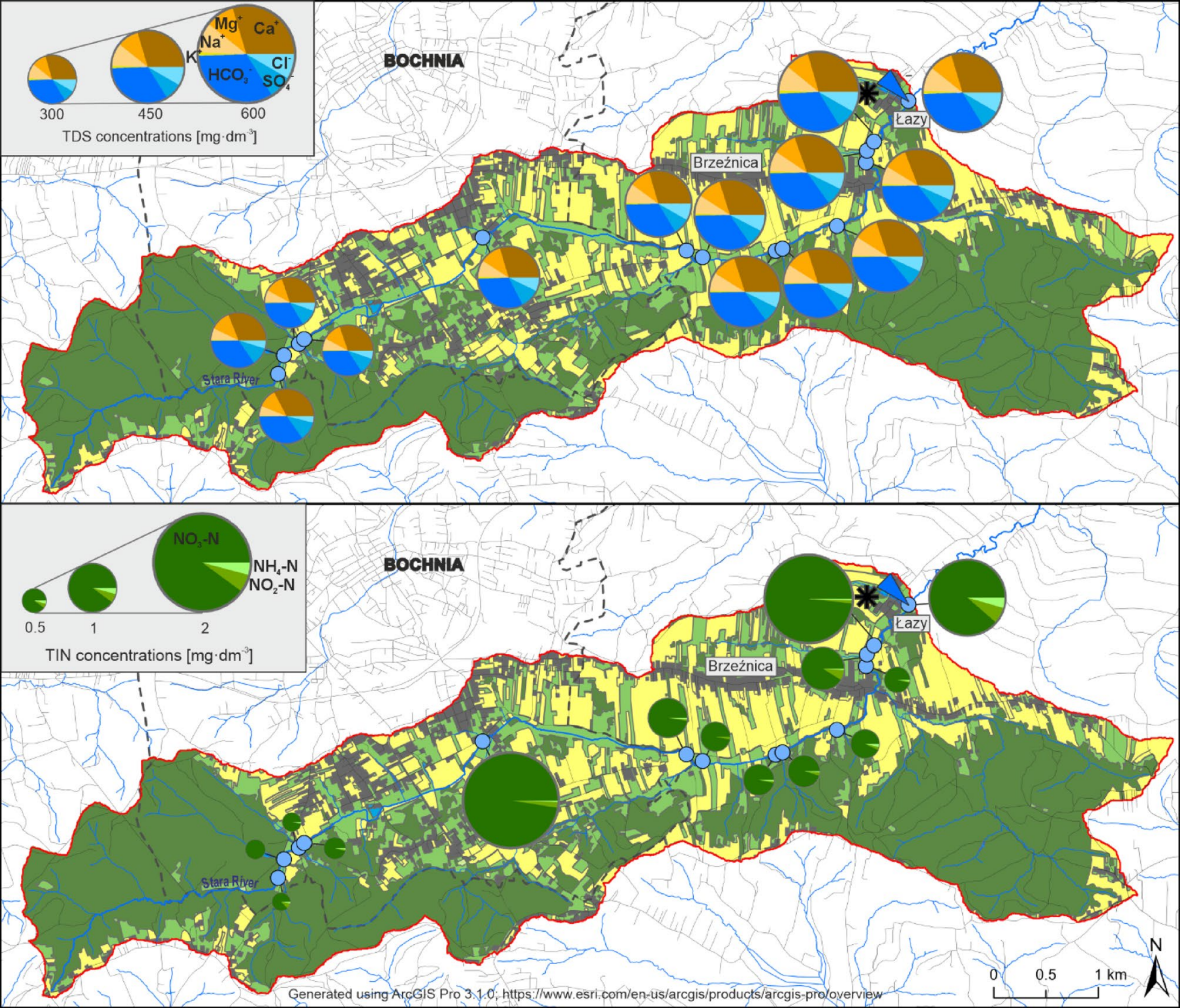
Changes in the structure of chemical composition, concentrations of water mineralization (TDS), nitrogen compounds (NH_4_-N, NO_3_-N, NO_2_-N) and concentrations of inorganic nitrogen (TIN) along the longitudinal profile of the Stara River. Values represent average concentrations and mean percentage contributions of chemical components from the years 2022–2024.

Along the course of the Stara River, an overall increase in the values of most physicochemical parameters is observed (Table 2). Electrical conductivity (EC), total dissolved solids (TDS), and the concentrations of major ions gradually increase downstream, although the magnitude of this increase does not exceed 50% over the studied section. In contrast, nutrient compounds – particularly nitrogen and phosphorus species – exhibit a substantially higher rate of increase. Specifically, the concentrations of NO_3_^-^, NH_4_^+^, and PO_4_^3-^ rise approximately 3-fold, 9-fold, and 90-fold, respectively. These sharp increases in nutrient concentrations are primarily associated with wastewater inputs and are most pronounced at sampling locations situated downstream of lateral tributaries and drainage canals that receive stormwater and domestic sewage from urbanized areas, as well as in the vicinity of intensive agricultural holdings. Additionally, a separate point source of nutrient enrichment is linked to the discharge from a small wastewater treatment plant located near the river channel. This study identifies both point and diffuse sources of pollution and emphasizes their influence on the longitudinal variability of water chemistry in the Stara River. In contrast to the notable increase in nutrient concentrations, only minor changes in SO_4_^2-^ concentrations are observed along the river course. The structural composition of dissolved ions along the river shows a somewhat different pattern. While the relative contributions of Ca^2+^, Mg^2+^, HCO_3_^-^, and SO_4_^2-^ decrease downstream, the proportions of Na^+^, Cl⁻, and K^+^ increase significantly – by 74%, 116%, and 131%, respectively (Fig. 2). A similar trend is observed in the relative shares of nutrient compounds: the proportions of NO_3_^-^, NH_4_^+^, and PO_4_^3-^ increase approximately 2-fold, 6-fold, and 40-fold, respectively. Nevertheless, it is important to emphasize that the absolute concentrations of nitrogen and phosphorus compounds in the Stara River remain low throughout the study period.

**Table 2:** The mean values and coefficients of variation (CV) of physicochemical water parameters along the longitudinal profile of the Stara river in 2022–2024.

Sampling point	Parameter	pH	EC	Ca^2+^	Mg^2+^	Na^+^	K^+^	NH_4_^+^	HCO_3_^−^	SO_4_^2−^	Cl^−^	NO_3_^−^	NO_2_^−^	PO_4_^3−^
		–	µS·cm^−1^	mg·dm^−3^										
1	Mean	6.99	414.3	59.00	12.35	11.79	1.70	0.01	194.56	47.37	11.82	1.48	0.14	0.01
	CV [%]	1.56	11.9	16.06	15.92	4.71	19.65	51.59	27.19	10.33	5.17	38.07	163.18	34.64
2	Mean	7.33	418.1	56.13	13.07	14.04	1.97	0.00	197.04	45.56	15.02	1.79	0.01	0.01
	CV [%]	1.14	9.4	13.51	14.51	5.09	16.45	43.80	24.54	11.20	4.46	23.39	142.84	54.23
3	Mean	7.29	394.4	51.66	12.18	14.70	1.95	0.01	173.45	42.99	15.45	1.72	0.01	0.01
	CV [%]	0.71	8.1	12.46	13.03	4.11	20.20	84.82	22.50	12.29	6.19	26.06	89.13	34.64
4	Mean	7.29	394.6	51.72	12.19	14.59	2.09	0.01	179.34	41.75	15.32	1.96	0.02	0.01
	CV [%]	1.27	7.5	11.82	12.57	2.64	18.75	114.36	21.59	12.59	6.25	28.43	131.93	34.64
5	Mean	7.23	487.9	61.17	14.20	20.16	4.80	0.01	199.35	49.35	25.30	8.50	0.13	0.03
	CV [%]	2.08	6.4	9.54	11.92	3.41	40.88	122.15	17.81	3.30	9.35	59.92	129.56	83.57
6	Mean	7.23	509.8	64.29	13.75	23.63	3.53	0.01	226.04	42.23	31.05	3.52	0.04	0.02
	CV [%]	0.96	2.8	8.38	10.85	17.55	25.50	106.80	20.63	10.74	33.65	58.29	144.73	118.68
7	Mean	7.37	539.0	69.66	15.63	22.82	3.50	0.01	249.98	42.73	30.93	2.57	0.07	0.01
	CV [%]	1.49	4.6	8.98	11.96	11.16	23.10	85.22	18.61	12.16	23.54	49.36	170.24	34.64
8	Mean	7.45	540.3	70.47	15.91	22.52	3.39	0.01	253.85	42.08	29.67	2.76	0.04	0.01
	CV [%]	1.50	4.5	9.34	11.79	8.44	25.35	102.53	19.28	10.48	21.27	60.03	167.72	39.46
9	Mean	7.48	541.3	70.85	15.92	22.62	3.38	0.01	246.98	41.26	28.77	2.84	0.05	0.01
	CV [%]	1.42	4.5	9.15	11.56	7.61	25.71	64.21	17.18	10.54	22.01	56.14	133.32	34.64
10	Mean	7.44	542.6	71.07	15.79	22.76	3.34	0.04	262.87	40.91	28.50	2.43	0.06	0.01
	CV [%]	1.63	4.7	9.16	11.91	4.82	25.71	128.47	18.65	11.78	16.70	53.06	133.56	34.64
11	Mean	7.53	555.5	74.96	15.30	22.38	4.18	0.01	259.95	43.69	27.41	2.26	0.04	0.01
	CV [%]	2.05	4.0	8.82	10.86	4.63	37.78	122.68	17.36	9.86	17.56	50.07	152.21	34.64
12	Mean	7.46	573.8	75.61	15.29	25.08	4.74	0.02	265.98	46.48	31.27	3.62	0.21	0.27
	CV [%]	2.34	6.6	9.43	11.41	17.39	49.13	139.70	18.92	10.29	15.06	48.64	163.14	124.63
13	Mean	7.40	608.1	77.55	15.50	31.22	6.30	0.01	267.43	49.12	38.23	7.95	0.06	1.49
	CV [%]	2.07	8.5	11.43	13.05	21.63	47.34	132.01	19.06	9.12	16.90	26.15	137.40	83.96
14	Mean	7.45	609.4	77.30	15.29	30.94	6.01	0.09	265.56	47.98	38.44	6.18	0.36	0.91
	CV [%]	3.19	10.7	970	10.50	31.24	54.38	104.52	17.93	12.07	25.16	29.84	160.31	93.61

### Land use changes in the Stara river catchment

**Fig. 3:**
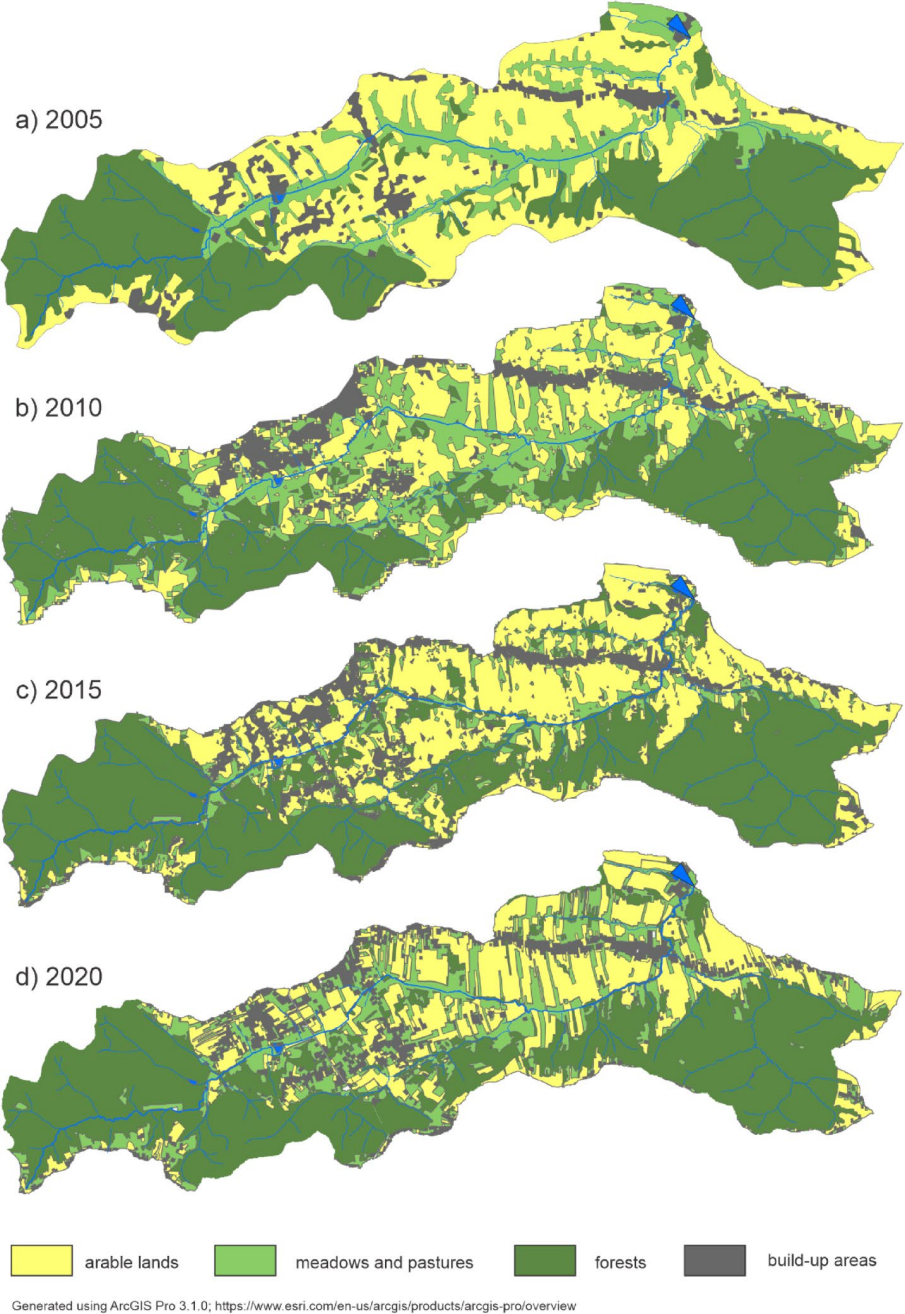
Land use changes in the Stara River catchment.

In the Stara River catchment, agricultural areas predominate, consisting mainly of arable land, which accounts for approximately 31%, and meadows and pastures, which make up 17%. A relatively large share of the catchment is covered by forests, around 45%. Built-up areas constitute the smallest share (Table 3; Fig. 3). During the study period, the share of agricultural land decreased by approximately 10%, mainly due to a decline in arable land (by about 12%). Over the years, the area of meadows and pastures increased. Additionally, the shares of built-up areas and forests grew by 3.6% and 6.3%, respectively (Table 3).

**Table 3:** Land use changes in the Stara river catchment.

Land use	2005		2010		2015		2020	
	km^2^	%	km^2^	%	km^2^	%	km^2^	%
Urban area	1.0	4.5	1.5	6.7	1.7	7.6	1.8	8.1
Forest	9.3	41.7	9.6	43.0	11.3	50.7	10.7	48.0
Arable land	8.1	36.3	6.0	26.9	6.8	30.5	5.4	24.2
Meadows and pastures	3.9	17.5	5.2	23.3	2.5	11.2	4.4	19.7
Total agricultural area	12.0	53.8	11.2	50.2	9.3	41.7	9.8	43.9

Arable land is primarily concentrated in the central and north-eastern parts of the catchment, characterized by gentler slopes; however, its area has significantly declined during the study period. The greatest increase in forest cover is observed in the highest-lying parts of the catchment, particularly in its southern section, where processes of secondary succession and afforestation contribute to improved water retention and reduced erosion. In contrast, the most substantial expansion of built-up areas occurred in the north-central part of the catchment, associated with the growth of the town of Bochnia and the urbanization of its suburban zone (Fig. 3).

### Long-term trends in water chemistry of the Stara river

The waters of the Stara River have a slightly alkaline pH, with an average electrical conductivity of 479 µS·cm⁻¹ and an average TDS value of 364 mg·dm⁻³ (Table 4). In the chemical composition of the water, HCO_3_^−^ ions dominate among anions, while Ca^2+^ ions prevail among cations. Among nitrogen compounds, NO_3_^−^ ions reach the highest concentrations. During the study period, nitrogen and phosphorus compounds exhibited the highest variability in water chemistry (CV > 200%). For major ions, variability was low to moderate, as the CV of concentration values generally did not exceed 35%, except for K⁺ ions.

**Table 4:** Descriptive statistics and coefficient of variation (CV) for physicochemical parameters and water discharge at the gauging station on the Stara river in the period 2003–2023.

Parameter	Unit	Mean	Min	Max	Std. Dev.	CV [%]
Q	m^3^·s^−1^	0.15	0.0001	64.09	0.86	569.9
pH	–	7.66	7.02	8.13	0.2	2.1
EC	µS·cm^−1^	479.2	129.2	800.0	103.9	21.7
TSS	mg·dm^−3^	152.9	0.2	4763.9	398.6	260.7
TDS		364.2	64.3	724.4	93.4	25.7
Ca^2+^		55.67	15.68	114.33	12.3	22.2
Mg^2+^		14.02	3.30	25.97	3.9	28.0
Na^+^		18.76	5.05	68.79	6.4	34.0
K^+^		8.83	1.44	50.21	10.5	119.2
NH_4_^+^		0.201	0.0004	3.559	0.4	193.2
HCO_3_^−^		186.36	30.55	425.80	55.9	30.0
SO_4_^2−^		55.80	18.54	120.00	15.3	27.3
Cl^−^		22.99	7.06	82.54	7.4	32.2
NO_3_^−^		7.17	0.005	28.56	5.2	72.1
NO_2_^−^		0.062	0.005	1.14	0.1	199.5
PO_4_^3−^		0.214	0.01	2.7	0.35	161.8
TIN		1.13	0.000	7.51	1.3	116.7

**Fig. 4:**
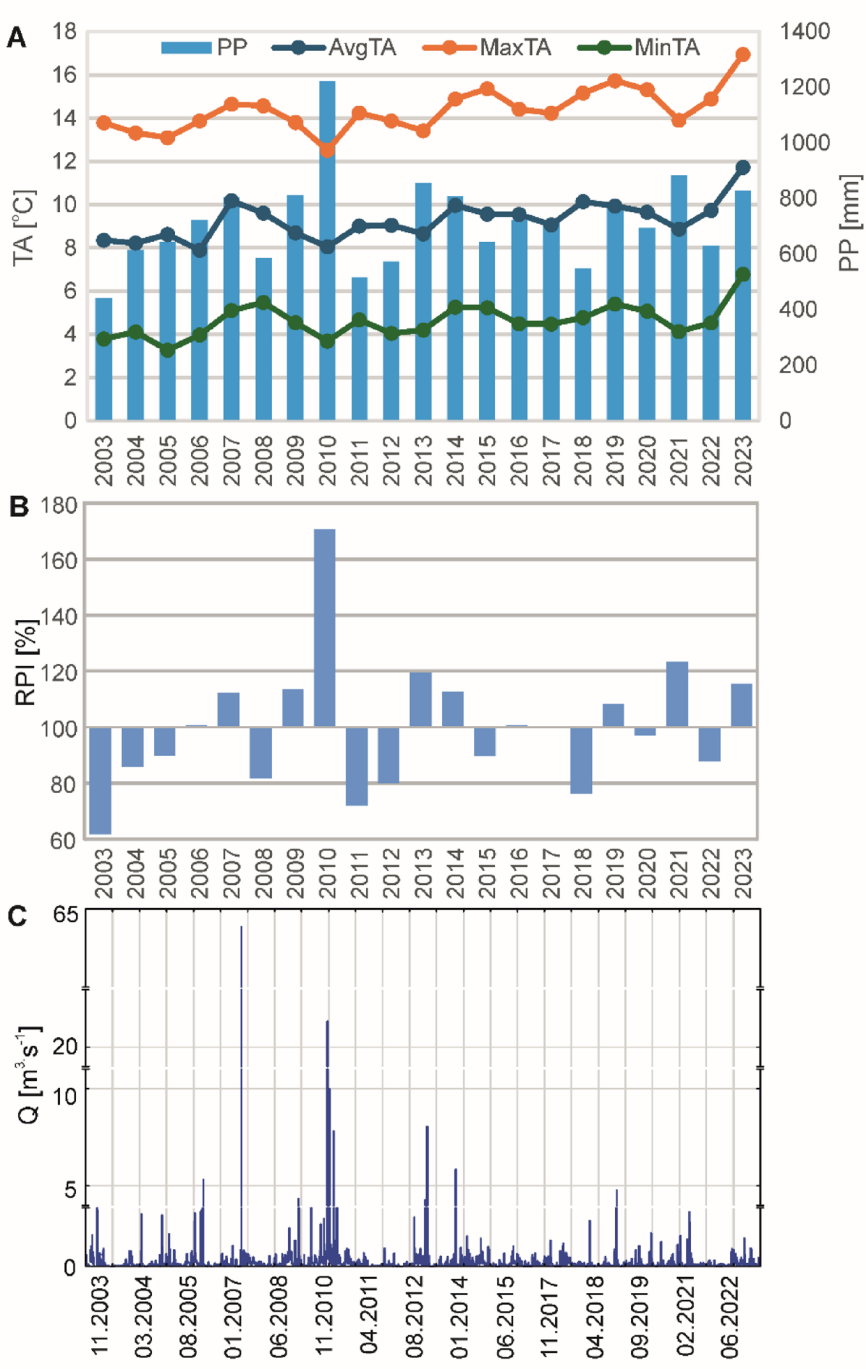
Climatic and hydrological variability in the Stara River catchment in the period 2003–2023. A – Trends in air temperature (AvgTA, MinTA, MaxTA) and precipitation (PP); B – Relative Precipitation Index (RPI); C – Hydrograph of the Stara River discharge.

**Fig. 5:**
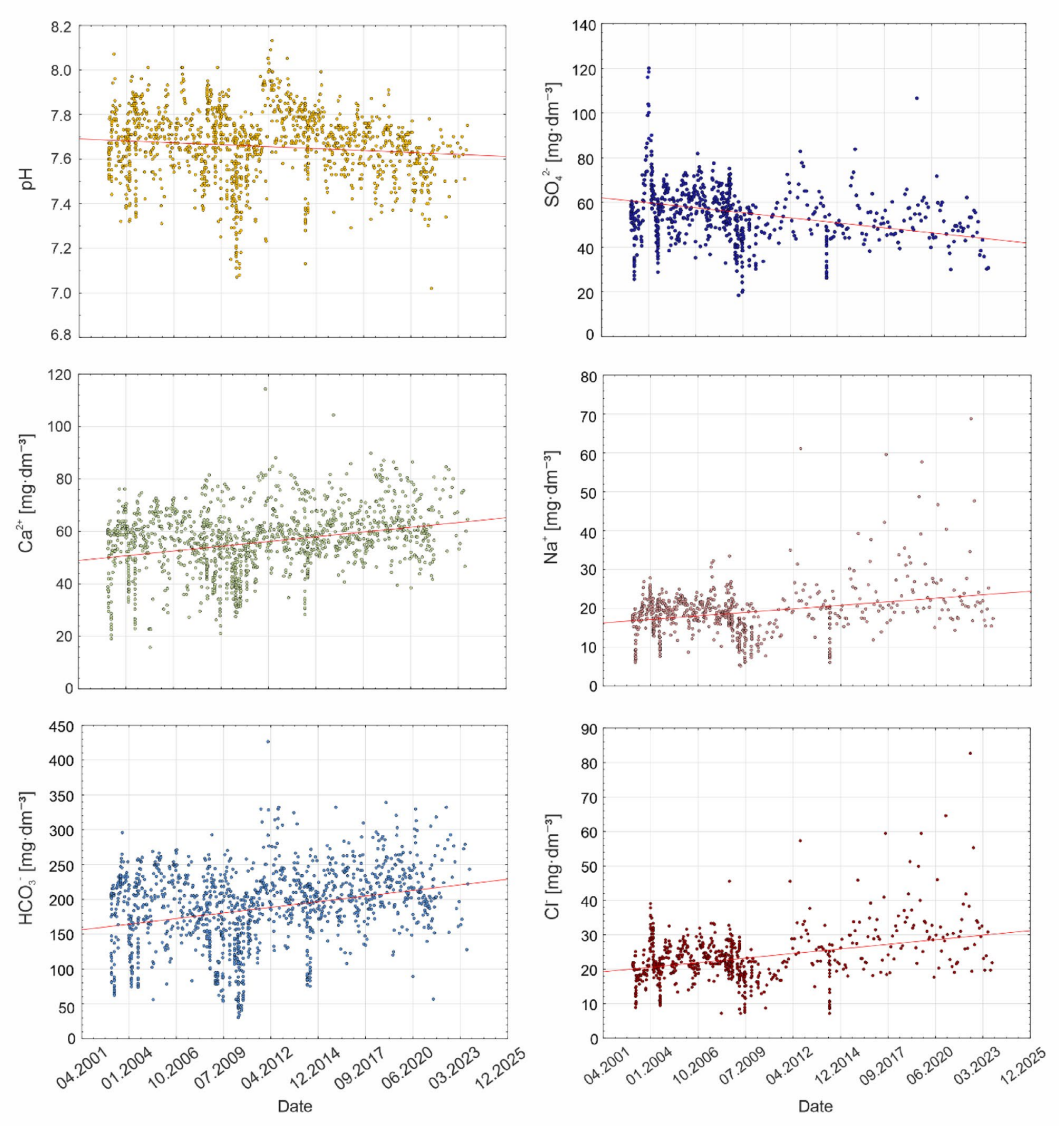
Significant annual trends in physicochemical parameters.

The results from Table 5; Fig. 4A show the long-term trends in water chemistry, hydrological, and meteorological parameters for the Stara River on an annual scale, as well as seasonally and during the growing and non-growing seasons. The results indicate a significant increase in air temperature in the Stara River catchment, with the most pronounced increase observed in MaxTA, rising by nearly 1 °C per decade. However, annual precipitation totals remain unchanged, and there are no significant trends in the discharge of the Stara River. nevertheless, the variability of these parameters during the study period and between individual years is often substantial, as indicated by the Relative Precipitation Index (Fig. 4B) and the hydrograph of the Stara River discharge (Fig. 4C). Significant annual trends in physicochemical parameters include pH, Ca^2+^, Na^+^, HCO_3_^−^, SO_4_^2−^ and Cl^−^. Water pH and SO_4_^2−^ concentrations show a significant decline, while Ca^2+^, Na^+^, HCO_3_^−^, and Cl^−^ concentrations are increasing (Fig. 5). The most substantial rise is observed for HCO_3_^−^, with an increase of nearly 24 mg·dm^−3^ per decade.

**Table 5:** Trend statistics for annual and seasonal water chemistry, hydrological and meteorological parameters.

Parameter	Year				Autumn				Summer				Spring				Winter				Non-growing				Growing			
	S	*p*	trend	Slope	S	*p*	trend	Slope	S	*p*	trend	Slope	S	*p*	trend	Slope	S	*p*	trend	Slope	S	*p*	trend	Slope	S	*p*	trend	Slope
pH	**−86**	**0.010**	↓	**−0.005**	−56	0.097	•	−0.006	**−100**	**0.003**	↓	**−0.008**	−44	0.194	•	−0.005	−8	0.833	•	−0.001	−20	0.566	•	−0.002	**−98**	**0.003**	↓	**−0.009**
EC	26	0.450	•	1.664	−14	0.695	•	−0.513	**72**	**0.032**	↑	**4.181**	24	0.487	•	1.125	−2	0.976	•	−0.044	16	0.651	•	0.704	50	0.139	•	2.305
TSS	−13	0.675	•	−2.464	27	0.363	•	1.122	−9	0.780	•	−0.891	−11	0.726	•	−0.663	23	0.441	•	1.411	−41	0.162	•	−1.451	15	0.624	•	1.334
TDS	62	0.065	•	3.713	6	0.880	•	0.690	**98**	**0.003**	↑	**5.705**	**68**	**0.043**	↑	**3.785**	52	0.124	•	2.295	**68**	**0.043**	↑	**2.870**	**70**	**0.037**	↑	**4.250**
Ca^2+^	**108**	**0.001**	↑	**0.621**	18	0.608	•	0.183	**106**	**0.002**	↑	**0.782**	**126**	**0.000**	↑	**0.817**	**84**	**0.012**	↑	**0.493**	**100**	**0.003**	↑	**0.622**	**98**	**0.003**	↑	**0.592**
Mg^2+^	−34	0.319	•	−0.055	−62	0.065	•	−0.181	18	0.608	•	0.028	−2	0.976	•	−0.015	−56	0.097	•	−0.133	−50	0.139	•	−0.068	−24	0.487	•	−0.048
Na^+^	**88**	**0.009**	↑	**0.438**	32	0.349	•	0.250	**82**	**0.014**	↑	**0.561**	**76**	**0.024**	↑	**0.315**	**82**	**0.014**	↑	**0.272**	**88**	**0.009**	↑	**0.332**	64	0.057	•	0.487
K^+^	−38	0.264	•	−0.052	−28	0.415	•	−0.098	0	1.000	•	−0.001	**−78**	**0.020**	↓	**−0.079**	−14	0.695	•	−0.023	−20	0.566	•	−0.035	−16	0.651	•	−0.028
NH_4_^+^	31	0.364	•	0.001	19	0.586	•	0.000	−24	0.486	•	−0.0004	23	0.506	•	0.0001	45	0.183	•	0.002	45	0.183	•	0.003	19	0.586	•	0.000
HCO_3_^−^	**84**	**0.012**	↑	**2.370**	26	0.450	•	0.628	**90**	**0.007**	↑	**3.054**	**90**	**0.007**	↑	**3.503**	**92**	**0.006**	↑	**2.564**	**98**	**0.003**	↑	**2.689**	**70**	**0.037**	↑	**1.986**
SO_4_^2−^	**−74**	**0.027**	↓	**−0.577**	**−72**	**0.032**	↓	**−0.751**	−60	0.075	•	−0.514	−48	0.156	•	−0.619	**−80**	**0.017**	↓	**−0.656**	−60	0.075	•	−0.426	**−66**	**0.050**	↓	**−0.461**
Cl^−^	**102**	**0.002**	↑	**0.573**	44	0.194	•	0.361	**104**	**0.002**	↑	**0.792**	**80**	**0.017**	↑	**0.524**	**88**	**0.009**	↑	**0.627**	**108**	**0.001**	↑	**0.540**	**82**	**0.014**	↑	**0.644**
NO_3_^−^	7	0.856	•	0.018	33	0.333	•	0.107	**83**	**0.013**	↑	**0.355**	−29	0.397	•	−0.135	35	0.304	•	0.189	1	1.000	•	0.000	51	0.130	•	0.186
NO_2_^−^	62	0.065	•	0.002	**87**	**0.007**	↑	**0.000**	10	0.772	•	0.000	64	0.054	•	0.000	**77**	**0.018**	↑	**0.000**	39	0.243	•	0.001	54	0.108	•	0.002
PO_4_^3−^	38	0.262	•	0.005	45	0.181	•	0.001	64	0.056	•	0.017	40	0.237	•	0.001	42	0.209	•	0.000	17	0.627	•	0.000	49	0.147	•	0.009
TIN	13	0.717	•	0.007	43	0.204	•	0.031	**73**	**0.029**	↑	**0.084**	−32	0.347	•	−0.036	31	0.364	•	0.036	1	1.000	•	0.000	63	0.061	•	0.048
PP	40	0.239	•	6.620	42	0.216	•	3.648	−4	0.928	•	−1.007	22	0.526	•	1.942	52	0.124	•	1.458	30	0.381	•	1.947	48	0.156	•	4.208
AvgTA	**94**	**0.005**	↑	**0.086**	**66**	**0.050**	↑	**0.053**	**72**	**0.032**	↑	**0.054**	4	0.928	•	0.005	**82**	**0.014**	↑	**0.190**	**96**	**0.004**	↑	**0.141**	34	0.319	•	0.018
MaxTA	**102**	**0.002**	↑	**0.096**	**66**	**0.050**	↑	**0.068**	**74**	**0.027**	↑	**0.073**	14	0.695	•	0.036	**82**	**0.014**	↑	**0.139**	**104**	**0.002**	↑	**0.148**	22	0.526	•	0.020
MinTA	**72**	**0.032**	↑	**0.057**	52	0.124	•	0.043	12	0.740	•	0.008	−32	0.349	•	−0.034	**72**	**0.032**	↑	**0.167**	**74**	**0.027**	↑	**0.110**	−16	0.651	•	−0.012
Q	−38	0.264	•	−0.003	32	0.349	•	0.001	−28	0.415	•	−0.001	−54	0.110	•	−0.007	−14	0.695	•	−0.001	−56	0.097	•	−0.004	−24	0.487	•	−0.002

S – Mann-Kendall S statistic, Slope – Sen’s slope estimator, p – significance level, ↓– deacreasing trend, ↑ – increasing trend, • – no trend. Statistically significant trends (*p* = 0.05) are highlighted in bold.

Summer exhibits the most significant changes in water chemistry, with increasing TDS, EC, dissolved ion concentrations (Ca^2+^, Na^+^, Cl⁻, HCO_3_^−^) and rising NO_3_^−^ and TIN levels. The highest increase is observed for TDS – approximately 57 mg·dm⁻³ per decade. The least significant trends in water chemistry are observed in autumn (SO_4_^2−^ – decreasing trend and NO_2_^−^ – increasing trend). In spring, significant increasing trends were found for TDS, Ca^2+^, Na^+^, HCO_3_^−^, and Cl^−^, whereas K^+^ concentrations in the Stara River decrease. In winter, an increase in the concentrations of Ca^2+^, Na^+^, HCO_3_^−^, and Cl^−^ ions is also observed over the studied period. Additionally, an increase in NO_2_^−^ concentrations and a decrease in SO_4_^2−^ were noted. Among the seasons, the greatest increasing trends in TDS and ion concentrations are observed in summer, with the exception of HCO_3_^−^, which shows the highest increase in spring. Spring stands out from other seasons due to the absence of significant air temperature trends. In the remaining seasons, a significant increasing trend is observed for AvgTA and MaxTA, while in winter, MinTA also shows a significant rising trend. Winter also exhibits the largest increases in air temperature values, with AvgTA rising by nearly 2 °C per decade. No significant trends are observed for PP and Q in any season. During the growing season, more significant trends in the water chemistry of the Stara River are observed compared to the non-growing season, but there are no significant trends in meteorological parameters. In contrast, during the non-growing season, air temperature, TDS, and the concentrations of Ca^2+^, Na^+^, HCO_3_^−^, and Cl^−^ increase significantly.

The relationships between the physicochemical parameters of water and land use for annual scale and for different seasons are summarized in Table 6. The strength of the relationships between catchment land use and river water chemistry is weak to moderate, with approximately half being statistically significant. The most negative correlations are observed between meadows and pastures and water chemistry. Only in the spring period do positive, though very weak (*r* < 0.2), correlations between water chemistry and meadows and pastures dominate.

On the other hand, the most positive correlations are found between water chemistry and forests. Only in spring do negative correlations prevail, and they are weaker. Positive correlations between biogenic compounds (N, P) and land use are observed in forested and urban areas, whereas agricultural land mostly shows negative correlations with the concentrations of these ions. It is noteworthy that SO_4_^2−^ is positively correlated only with arable land and total agricultural area. Most major ions, especially Ca^2+^, Mg^2+^, and HCO_3_^−^, as well as TDS, show a weak relationship with land use. The strength of this relationship slightly increases during winter and autumn.

**Table 6:** Pearson correlation between the physicochemical characteristics of water and land use in the Stara river catchment.

Parameter	Arable land	Forest	Agriculture area	Meadows & Pastures	Urban	Arable land	Forest	Agriculture area	Meadows & Pastures	Urban	Arable land	Forest	Agriculture area	Meadows & Pastures	Urban	Arable land	Forest	Agriculture area	Meadows & Pastures	Urban	Arable land	Forest	Agriculture area	Meadows & Pastures	Urban
pH	−0.01	0.01	−0.01	0.01	0.02	**0.22**	**−0.20**	**0.23**	0.01	**−0.26**	**0.35**	−0.10	**0.16**	**−0.27**	**−0.28**	−0.03	−0.09	0.06	0.12	0.00	**0.15**	**−0.11**	**0.13**	−0.03	**−0.15**
EC	**0.32**	0.05	0.03	**−0.39**	**−0.21**	−0.02	**−0.17**	**0.16**	**0.18**	−0.10	0.11	**0.18**	−0.13	**−0.29**	−0.01	**0.32**	0.04	0.04	**−0.37**	**−0.22**	**0.10**	−0.01	0.04	**−0.09**	**−0.09**
TDS	**0.19**	**0.27**	**−0.19**	**−0.49**	−0.02	−0.09	0.03	−0.04	0.05	0.05	0.00	**0.32**	**−0.27**	**−0.29**	0.14	**0.26**	0.14	−0.06	**−0.40**	−0.14	0.03	**0.14**	**−0.11**	**−0.17**	0.03
TSS	**−0.62**	**0.42**	**−0.49**	**0.21**	**0.62**	**−0.25**	**0.32**	**−0.34**	−0.10	**0.34**	**−0.32**	0.14	**−0.19**	**0.21**	**0.29**	**−0.58**	**0.24**	**−0.34**	**0.38**	**0.53**	**−0.36**	**0.26**	**−0.31**	**0.11**	**0.38**
Ca^2+^	−0.03	**0.48**	**−0.42**	**−0.47**	**0.23**	**−0.30**	**0.27**	**−0.29**	−0.01	**0.32**	**−0.27**	**0.41**	**−0.42**	−0.10	**0.37**	0.06	**0.30**	**−0.25**	**−0.34**	0.08	**−0.18**	**0.32**	**−0.31**	**−0.13**	**0.27**
Mg^2+^	**0.48**	−0.18	**0.26**	**−0.31**	**−0.42**	0.04	**−0.25**	**0.24**	**0.21**	**−0.17**	**0.40**	−0.12	**0.20**	**−0.31**	**−0.35**	**0.66**	**−0.26**	**0.39**	**−0.44**	**−0.61**	**0.32**	**−0.20**	**0.25**	**−0.12**	**−0.33**
Na^+^	**0.21**	**0.25**	−0.17	**−0.48**	−0.05	−0.09	−0.06	0.05	**0.14**	0.00	−0.01	**0.29**	**−0.25**	**−0.26**	0.12	0.09	**0.19**	−0.14	**−0.27**	0.00	−0.01	**0.11**	**−0.09**	**−0.11**	0.04
K^+^	**0.43**	**−0.28**	**0.34**	−0.15	**−0.43**	**0.40**	**−0.33**	**0.37**	−0.03	**−0.44**	**0.31**	**−0.24**	**0.28**	−0.10	**−0.33**	**0.34**	**−0.24**	**0.29**	−0.13	**−0.35**	**0.33**	**−0.29**	**0.32**	−0.05	**−0.37**
HCO_3_^−^	0.02	**0.36**	**−0.30**	**−0.39**	0.14	**−0.16**	0.14	**−0.15**	0.00	**0.16**	0.03	**0.32**	**−0.27**	**−0.33**	0.12	**0.25**	**0.19**	−0.10	**−0.43**	−0.11	−0.01	**0.20**	**−0.17**	**−0.18**	**0.09**
SO_4_^2−^	**0.58**	**−0.22**	**0.31**	**−0.38**	**−0.50**	**0.22**	**−0.37**	**0.38**	**0.18**	**−0.35**	**0.18**	−0.14	**0.16**	−0.06	**−0.19**	**0.20**	**−0.27**	**0.28**	0.04	**−0.26**	**0.25**	**−0.25**	**0.27**	0.00	**−0.29**
Cl^−^	0.12	**0.31**	**−0.24**	**−0.45**	0.04	−0.09	−0.13	0.10	**0.20**	−0.03	**−0.19**	**0.37**	**−0.36**	−0.15	**0.29**	−0.06	**0.22**	**−0.20**	−0.14	0.12	**−0.09**	**0.13**	**−0.13**	−0.04	**0.11**
NH_4_^+^	0.03	0.14	−0.11	−0.18	0.04	**0.27**	**−0.16**	**0.19**	−0.08	**−0.26**	0.02	−0.10	0.09	0.07	−0.05	**−0.17**	0.07	−0.09	0.10	0.13	0.08	−0.05	0.06	−0.03	**−0.08**
NO_3_^−^	**−0.53**	**0.49**	**−0.54**	0.04	**0.59**	**−0.27**	**0.38**	**−0.39**	**−0.14**	**0.37**	**−0.54**	**0.46**	**−0.52**	0.14	**0.59**	**−0.39**	**0.56**	**−0.58**	−0.11	**0.53**	**−0.39**	**0.45**	**−0.48**	−0.05	**0.49**
NO_2_^−^	0.07	0.08	−0.05	−0.16	−0.03	**0.33**	**−0.15**	**0.20**	**−0.14**	**−0.31**	**0.28**	−0.15	**0.20**	−0.15	**−0.27**	−0.03	**0.20**	**−0.17**	**−0.16**	0.08	**0.20**	−0.04	**0.09**	**−0.16**	**−0.18**
PO_4_^3−^	0.07	**0.37**	**−0.31**	**−0.45**	0.13	**0.16**	**0.36**	**−0.29**	**−0.51**	0.08	−0.06	**0.41**	**−0.37**	**−0.31**	**0.22**	−0.06	**0.55**	**−0.49**	**−0.44**	**0.28**	0.05	**0.39**	**−0.34**	**−0.42**	**0.15**
TIN	**−0.51**	**0.47**	**−0.52**	0.04	**0.57**	**−0.25**	**0.36**	**−0.37**	−0.14	**0.36**	**−0.51**	**0.43**	**−0.49**	0.14	**0.56**	**−0.38**	**0.55**	**−0.56**	−0.10	**0.52**	**−0.37**	**0.43**	**−0.46**	−0.05	**0.46**
Season	Winter					Spring					Summer					Autumn					Year				

In bold - statistically significant correlation coefficients (*p* = 0.05). Interpretation of Pearson correlation coefficients is based on the following scale: |r| < 0.2 – very weak or no correlation; 0.2 ≤ |r| < 0.4 – weak corelation; 0.4 ≤ |r| < 0.7 – moderate correlation; 0.7 ≤ |r| < 0.9 – strong correlation; |r| ≥ 0.9 – very strong correlation.

### Factors influencing water chemistry of the Stara river

**Fig. 6:**
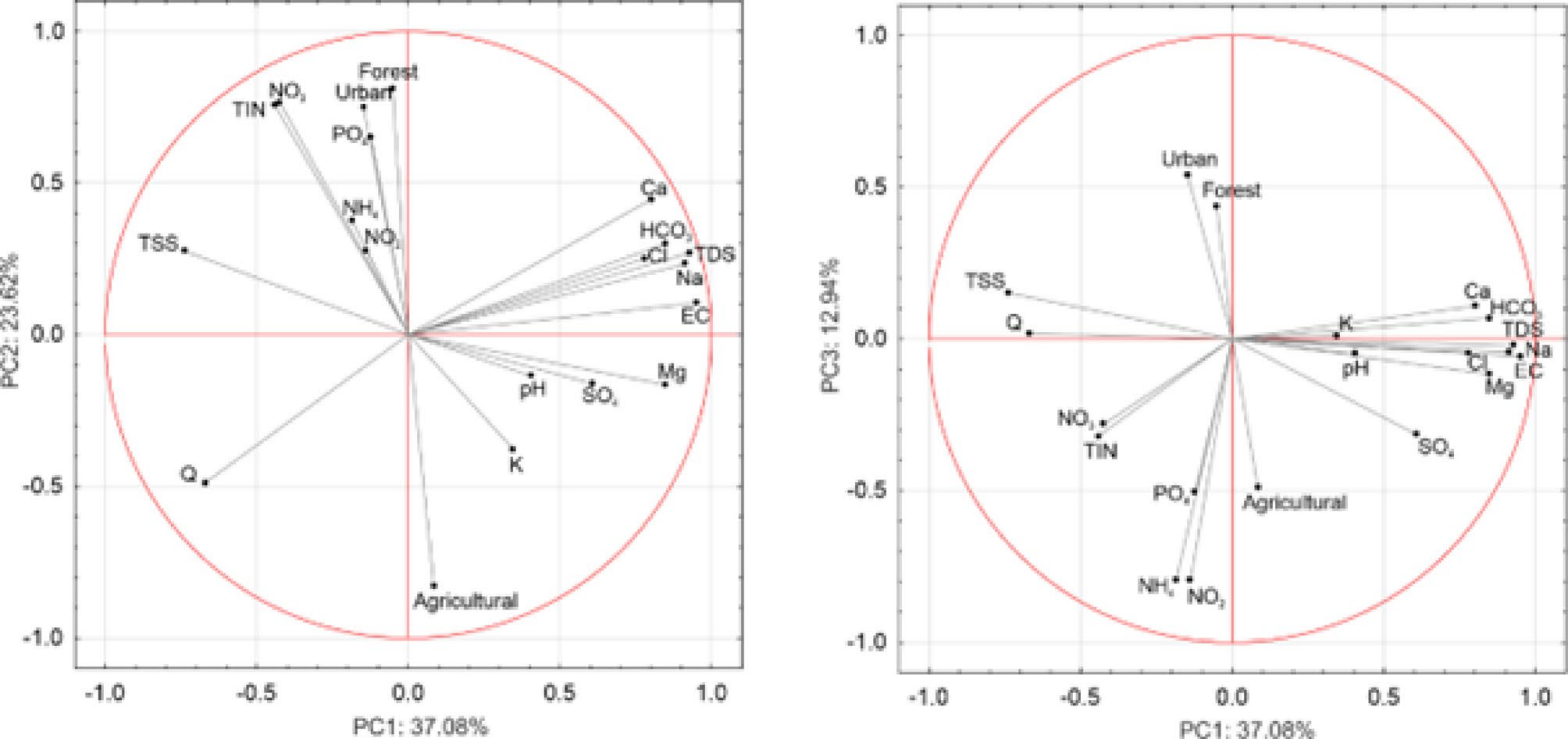
Principal Component Analysis of water chemistry and land use: the projection of factor loadings for PC 1 × 2 and PC 1 × 3.

The principal component analysis (PCA) identified three main factors shaping the water chemistry of the Stara River, collectively explaining 73.6% of the variability. Factor 1 accounted for 37.1% of the variability, factor 2 for 23.6%, and factor 3 for 12.9% (Fig. 6; Table 7). In factor 1, a relationship was observed between discharge, total suspended solids concentration, major ion concentrations, electrical conductivity, and total dissolved solids in the Stara River. A pattern emerged where higher discharge corresponded to increased total suspended solids in river water but lower concentrations of major ions. Factor 2 highlighted the relationship between land use, Q, and the concentrations of NO_3_^-^, PO_4_^3-^, and total inorganic nitrogen. The higher the share of urbanized and forested areas, the higher the values of biogenic compounds (NO_3_^-^, PO_4_^3-^, TIN), while Q was lower and there was less agricultural land. Factor 3 revealed a correlation between the share of agricultural and urbanized areas and the concentrations of NH_4_^+^, NO_2_^-^, and PO_4_^3-^. A higher share of agricultural land in the catchment was associated with a lower share of built-up areas and with increased concentrations of nitrogen (NH_4_^+^, NO_2_^-^) and phosphorus compounds in the river water.

**Table 7:** Principal component analysis (PCA) of water chemistry and land use: eigenvalues, explained variance and cumulative explained variance.

Parameter	Factor 1	Factor 2	Factor 3
Eigenvalue	7.9	4.2	2.7
Variance [%]	37.1	23.6	12.9
Cumulative variance [%]	37.1	60.7	73.6

## Disscusion

As water travels downstream, its chemistry can change due to various factors, such as tributary inflows, groundwater interactions, and anthropogenic inputs. These changes may manifest as either smooth gradients or abrupt shifts, depending on specific conditions and influences. Nevertheless, the study by Hensley et al.^37^ also demonstrated that longitudinal profiles of different physicochemical water parameters exhibit distinct patterns, resulting from spatial and temporal variations in the factors and processes that shape them. Along the longitudinal profile of the Stara River, it is evident that nutrient compounds (N and P) are influenced not only by natural processes but also predominantly by anthropogenic factors, as indicated by abrupt, stepwise changes in both their concentrations and shares. Among the major ions, Na^+^ and Cl^-^ are also subject to anthropogenic influences, as their concentrations and relative contributions to the chemical composition increase significantly, whereas the proportions of Ca^2+^, Mg^2+^, and HCO_3_^-^ decrease.

The impact of land use changes within the catchment is also noticeable. In the upper course of the river, within the forested area, the increase in TDS and ion concentrations in the river water is relatively small. However, as land use transitions to agricultural and urban areas, these parameter increases become markedly more pronounced. Many studies show that human activities, such as agriculture, industrial discharges, and urbanization, contribute to the chemical composition of river water. These activities can introduce pollutants such as heavy metals, nutrients, and organic matter, which can degrade water quality^38–40^.

The analysis indicates that climate change is occurring in the Stara River catchment, evident in rising air temperatures, both annually and in specific seasons, including the non-growing season. However, there are no significant trends in total precipitation, either on an annual scale or in individual seasons. Despite increased evaporation from rising air temperatures, the lack of significant changes in annual or seasonal precipitation has resulted in no notable changes in river discharge. However, most studies suggest that climate change affects river chemistry primarily through discharge variations driven by changes in precipitation^2^. A decrease in river discharge, caused by reduced precipitation and rising air temperatures, increases the concentration of most ions in river water due to reduced dilution capacity^2,41^. Conversely, an increase in river discharge induced by climate change creates a dilution effect, reducing the concentration of chemical substances, particularly major ions^42^. Nevertheless, higher discharge can also lead to increased transport of suspended solids and higher concentrations of biogenic substances (N, P, C). This results from more intense and frequent surface runoff, particularly from agricultural areas or impervious urban surfaces^43^. Climate change accelerates also chemical weathering of rocks, which releases main ions into river systems. This process is driven by increased temperatures and changes in precipitation patterns, which enhance the dissolution of minerals. The study by Gong et al.^44^ found that between 1980 and 2020, the largest proportion of ions in river waters derived from rock weathering consisted of HCO_3_⁻ and Ca^2^⁺, and the rate of weathering has increased by 30%. Similarly, in the Stara River catchment, the highest annual increasing trend is observed for the concentration of these ions, which may originate from the thick loess deposits present in the area.

However, climate change associated solely with a significant increase in air temperature also influences the chemistry of surface waters. Rising air temperatures affect water temperature, which in turn alters biogeochemical balances in aquatic ecosystems. This impacts gas solubility, the intensity of biological processes, and the transport and retention of chemical components. For example, studies by Ducharne^45^ on the Seine River (France) showed that a warming climate leads to increased oxygen deficits in water and higher phytoplankton biomass. Additionally, climate change-induced temperature increases significantly affect nitrogen dynamics. Higher temperatures can enhance denitrification processes, which reduce nitrate concentrations in water. This was demonstrated in studies by Gervasio et al.^46^where an increase in average air temperature by approximately 3 °C over 30 years led to a 30% reduction in total nitrogen loads in the Po River. This resulted from boosted denitrification capacity of river sediments along the lowland reaches, particularly during the summer period.

Land use changes significantly impact river water chemistry, with various thresholds indicating when these impacts become important. For example, a minimum of 45% natural vegetation cover is required to maintain nitrogen concentrations within acceptable levels in river waters^47^. On the other hand, studies by Kim et al.^48^ indicate that not exceeding 10% of impermeable surfaces in a catchment is crucial to mitigate the degradation of water quality. Increased residential and urban land use strongly correlates with deteriorating water quality, particularly in terms of higher concentrations of pollutants such as nitrogen, phosphorus, and carbon compounds^49–51^. In the Stara River catchment, a deterioration in water quality is also observed as the built-up area expands. This is evident in the spatial variation of water chemistry along the river course, particularly through point-source wastewater discharges, which cause abrupt changes in nitrogen and phosphorus compounds. This impact is also reflected in the increasing trends of Na^**+**^ and Cl^**-**^ ion concentrations. Rising river water salinity is a significant issue observed worldwide^52^. The increase in water salinity associated with urbanization is influenced by urban expansion, the increase in impervious surfaces, the application of road salts, and the impact of wastewater treatment plants^53^. Also agricultural activities contribute significantly to nutrient loading in rivers, particularly nitrates and phosphates, which can lead to eutrophication in surface waters^54,55^. The decline in arable land and the increase in meadows and pastures in the Stara River catchment have a positive effect on water quality, as evidenced by the mostly insignificant long-term trends for N and P compounds (Table 5). A decreasing trend in SO_4_^2-^ concentrations is also observed in the Stara River catchment, which results from the reduction in agricultural land use and the decrease in fertilizer application, as fertilizers are an important source of sulfur in river waters. The impact of agricultural land use changes on SO_4_^2-^ concentrations is most pronounced in winter, as confirmed by trends and correlation coefficients (Tables 5 and 6). Nevertheless, long-term observations also indicate that the concentrations of ions such as SO_4_^2-^ and NO_3_⁻ in river water have decreased in some regions due to reduced acidic deposition. The decline in acid deposition is also observed in the Carpathians; for example, the study by Minďaš et al.^56^ showed a downward trend in S-SO_4_ concentrations in precipitation at Chopok, with a rate of **−** 0.0567 mg·dm⁻³ per year.

Most research indicate that forest land acts as a natural water purifier. Catchment with higher forest cover usually have better water quality^57,58^. The observed increase in forested area in the Stara River catchment is an important factor positively influencing river water quality. As confirmed by the spatial variability analysis, the upper section of the Stara River is characterized by the lowest TDS and concentrations of most ions, including nutrients. Additionally, the presence of forests in the uppermost section of the river plays a crucial role in hydrological regulation, erosion control, water retention, and the improvement of surface water quality^59^. By stabilizing slopes in the highest parts of the Stara River catchment, forests reduce water erosion, thereby decreasing the amount of transported sediments. This is further supported by the lack of significant trends in suspended solids concentrations.

Principal Component Analysis (PCA) revealed that hydrometeorological conditions are the most important factors shaping the concentrations of major ions, TSS, and TDS in the waters of the Stara River. As discharge increases, the concentrations of major ions decrease due to the dilution effect. In contrast, during drought periods with low discharge, concentration effects occur, leading to an increase in major ion concentrations. The opposite relationship is observed for total suspended solids (TSS) – during flood events, TSS values increase, whereas during droughts, they decrease. However, factors 2 and 3 are associated with the impact of land use on nutrient concentrations in the river. Factor 2 indicates that the observed increase in urban and forested areas within the catchment leads to higher nitrogen (N) and phosphorus (P) concentrations in river waters. Studies confirm that the expansion of residential and urban land deteriorates water quality, partly due to the increased input of nitrogen and phosphorus compounds^60^. A similar trend is observed in the Stara River catchment, where the built-up area is expanding. Additionally, the impact of human activities on rising nutrient concentrations has been confirmed by the spatial variability analysis of water chemistry along the river course. However, most studies indicate that a larger forested area generally improves surface water quality^61^. Nevertheless, during the autumn-winter period, when trees shed leaves and nitrogen uptake by plants slows down, NO_3_⁻ runoff into rivers may increase^62^. These processes are particularly intensive after heavy rainfall and during spring snowmelt, when water leaches nitrogen accumulated in the soil during winter^63^. Indeed, higher correlation coefficients between forested areas and nitrogen compounds are observed in the Stara River waters during winter and autumn (Table 6). In Turn, Factor 3 highlights the impact of agriculture on water chemistry, primarily on the concentrations of nutrient compounds (NH_4_^+^, NO_2_^−^, and PO_4_^3−^). The analysis confirmed the observed trend in the Stara River catchment, showing that as forest and built-up areas expand, the share of agricultural land decreases, leading to a reduction in nitrogen (N) and phosphorus (P) concentrations in river water.

## Conclusion

Ongoing climate change, combined with changes in land use within the catchment area, has a significant impact on the chemical composition of river water. These changes affect both long-term trends in water quality and the spatial variability of the chemical composition within the catchment. The combined influence of these two factors leads to significant modifications in the chemical composition of the water, which is reflected both in the temporal scale (long-term changes in the concentrations of individual compounds) and the spatial scale (differences in water chemistry in various parts of the catchment). The rise in air temperature in the Stara River catchment, especially during the winter period, leads to the intensification of biogeochemical processes in the catchment and alters the weathering rate. This, in turn, results in an increase in mineralization and the concentration of major ions, particularly bicarbonates and calcium, in the river water. Changes in land use have a significant impact on the spatial variability of water chemistry within the catchment. The diversity of land use in the Stara River catchment, which includes agricultural, forested, and urbanized areas, leads to distinct differences in water quality in different parts of the catchment. Nevertheless, long-term trends in water chemistry also indicate the influence of land use changes, primarily through the increasing salinity of the Stara River water. The reduction in agricultural land in favor of forested areas and permanent grasslands has had a positive effect on the water quality of the Stara River, resulting in decreased concentrations of nitrogen, phosphorus, and sulfates. In contrast, the expansion of built-up areas and anthropogenically transformed land has had a negative impact, contributing to local deterioration in water quality, as indicated by increased electrical conductivity and elevated concentrations of chlorides and nutrient-related ions. Ongoing changes in the water chemistry of the Stara River are similarly influenced by both rising temperatures driven by global warming and land cover changes within the catchment.

## Data Availability

The datasets generated and analyzed during the current study are available from the corresponding author upon request.
